# Grand challenges for cheminformatics

**DOI:** 10.1186/1758-2946-1-1

**Published:** 2009-03-17

**Authors:** David J Wild

**Affiliations:** 1 Editor-in-Chief, Journal of Cheminformatics, Indiana University School of Informatics, Bloomington, IN 47408, USA

## Editorial

Welcome to the *Journal of Cheminformatics*. We are proud to be associated with a field that has a history longer than most applied computational disciplines; that has elegantly solved so many basic (and not so basic) problems; that has a reputation for intellectual rigor and good-naturedness; that has hundreds of scholarly articles published; and that has impacted fields as diverse as drug discovery, library science and database searching. Every time an HIV patient takes a life-saving protease inhibitor, a synthetic chemist finds a needed journal article through a substructure search, or a medicinal chemist finds a new, promising set of compounds through a virtual screening experiment, they have good reason to thank the practitioners of the field we now call cheminformatics.

But therein lies a problem. We have impacted a diverse group of people and domains that defy a single categorization. We have done so with a small, scattered academic presence in very different environments (current cheminformaticians sit with various levels of comfort in Information Studies, Informatics, Computer Science, Chemistry, and Pharmacy departments among others). We only settled on a name for the field less than a decade ago, and we still struggle with its spelling. Much research has been carried out in pharmaceutical companies or with industry support, so is not as visible or accessible as comparable research in related fields such as bioinformatics. Unlike biology, academic chemistry traditionally has a much different scope than industrial and pharmaceutical chemistry.

So are we all about Library & Information Science or Computational Drug Discovery? Is our work theoretical or pragmatic? Are our "customers" biologists or chemists? Do we have an academic or industry focus? Historically, the answer to these questions is "both". Those of us who care about the future of this field need to honestly ask whether cheminformatics is a bona-fide discipline in its own right, or whether it is a loose collection of practitioners without a single, definable focus. How we answer this question will determine the approach we should take to research, funding, publication, and education in the future.

We firmly believe that Cheminformatics needs to be centered and cemented as a true, distinct discipline, with explicit funding support, dedicated journals and conferences, more visibility in the academic community, strengthened ties to related fields, and a mechanism to educate the next generation of researchers. To maximize our chances of this happening, we need to be clear about what we can contribute to the world in the future, we need to apply ourselves to some of the grand challenges of the 21^st ^century, and we need to find our place in relation to our sister field of bioinformatics. We can no longer point backwards to our prior successes, and potential marginal improvements on our successes, but must as a community prioritize our efforts for the future and change some our strategies for research and education accordingly.

We identify four "grand challenge" areas that we think should be an important focus for cheminformatics.

### Overcoming stalled drug discovery

After the impressive successes in drug discovery toward the end of the last century, productivity in the pharmaceutical industry has declined as expenses have gone up. Cheminformatics can help by enabling fast, cheap virtual experiments to prioritize real experiments. As more drug discovery research is carried out in academia, institutes and small companies, and solutions will require pieces from cheminformatics, bioinformatics and other disciplines, cheminformatics knowledge and tools should be made as widely available as possible.

### Green chemistry & global warming

Global warming and preserving the environment will be one of the biggest challenges for mankind this century. Fundamental to this will be finding chemicals which are less polluting or less toxic to the environment, or improving chemical use to minimize environmental impact (e.g. in petrochemicals). Cheminformatics already has much to offer through computational toxicology and predictive modeling.

### Understanding life from a chemical perspective

Chemicals are being found to be increasingly important in cellular functions, for example through small molecule modulators and epigenetics. This has led to fields such as chemical biology, and more recently systems chemistry [1] and systems chemical biology [2], which seek to understand biological systems from a chemistry perspective. Integration of cheminformatics and bioinformatics methods will be key to this.

### Enabling the network of the world's chemical and biological information to be accessible and interpretable

We have seen huge leaps forward in the provision of freely accessible chemical databases such as PubChem [3] and ChemSpider [4]. A wealth of information is buried in these databases as well as many other related sources. Increasingly, this information is linked to biological information (such as targets, genes, experiments) and scholarly or informal publications, which opens up huge possibilities for data mining. Cheminformatics could potentially make all of this information very useful.

But of course these are just our thoughts. We need a discussion about priorities, and we need high quality research to point us in the right direction. Our aim in launching the *Journal of Cheminformatics *is to be a small but significant part in this process. Our name reflects our commitment to cheminformatics as a distinct discipline. Because we are an Open Access journal, our published work is immediately accessible through the web in a form which encourages propagation into other disciplines (such as being referenced in blog entries). We stand on the shoulders of BioMed Central, which contains some of the most highly cited journals in related fields such as Bioinformatics. We are committed to publishing only the highest quality research and insightful opinion that is most relevant to 21^st ^century science.

So welcome to the journal. In an editorial, we have the luxury of airing grand ideas but they may only be realized through the hard work of authors committed to this vision. So we thank the authors of articles already contributed, and we encourage you to help make the *Journal of Cheminformatics *a vibrant continuation into the 21^st ^century of a very important discipline.
